# Low frequency of somatic mutations in the *FH*/multiple cutaneous leiomyomatosis gene in sporadic leiomyosarcomas and uterine leiomyomas

**DOI:** 10.1038/sj.bjc.6600502

**Published:** 2002-08-12

**Authors:** K T Barker, S Bevan, R Wang, Y-J Lu, A M Flanagan, J A Bridge, C Fisher, C J Finlayson, J Shipley, R S Houlston

**Affiliations:** Section of Cancer Genetics, Haddow Laboratories, Institute of Cancer Research, 15 Cotswold Road, Sutton, Surrey SM2 5NG, UK; Section of Molecular Carcinogenesis, Institute of Cancer Research, Sutton, Surrey SM2 5NG, UK; Department of Pathology, University of Nebraska Medical Center, Omaha, Nebraska, USA; Department of Histopathology, Royal Marsden Hospital, London, UK; Department of Histopathology, St. George's Hospital Medical School, London, UK; Department of Histopathology, Royal Free and University College London Medical School, London, UK

**Keywords:** fumarate hydratase, mutation, leiomyomas, leiomyosarcomas

## Abstract

Germline mutations in the fumarate hydratase gene at 1q43 predispose to dominantly inherited skin and uterine leiomyomata and leiomyosarcomas. The enzyme, which is a component of the tricarboxylic acid cycle, acts as a tumour suppressor. To evaluate fumarate hydratase in respective sporadic tumours, we analysed a series of 26 leiomyosarcomas and 129 uterine leiomyomas (from 21 patients) for somatic mutations in fumarate hydratase and allelic imbalance around 1q43. None of the 26 leiomyosarcomas harboured somatic mutations in fumarate hydratase. Fifty per cent of leiomysarcomas tested showed evidence of allelic imbalance at 1q, but this was not confined to the vicinity of fumarate hydratase. Only 5% (seven out of 129) of the leiomyomas showed allele imbalance at 1q42-q43 and no somatic mutations in fumarate hydratase were observed. Our findings indicate that mutations in fumarate hydratase do not play a major role in the development of sporadic leiomyosarcomas or uterine leiomyomas.

*British Journal of Cancer* (2002) **87**, 446–448. doi:10.1038/sj.bjc.6600502 www.bjcancer.com

© 2002 Cancer Research UK

## 

Leiomyosarcomas of soft tissues are rare malignant tumours that have the phenotypic features of smooth muscle differentiation. They develop principally in adults and in a range of body sites, particularly the retroperitoneum, superficial soft tissues, and deep compartments of the extremities (Enzinger and Weiss, 1995; Shipley, 2002). Karyotypically leiomyosarcomas are characterised by a high-degree of chromosome instability and a wide range of anomalies (Packenham *et al*, 1997; Wang *et al*, 2001; Shipley, 2002). In contrast their benign counterpart – leiomyomas – are common, especially uterine fibroids which are the most common tumours in women during their reproductive years (Stewart, 2001). Furthermore, leiomyomas have been reported to carry few genetic changes (Packenham *et al*, 1997; Mao *et al*, 1999).

Little is known about the molecular basis of sporadic leiomyosarcomas or leiomyomas. We have, however, recently shown that germline mutations in the fumarate hydratase (*FH*) gene at chromosome 1q43 cause dominantly inherited skin and uterine leiomyomata and leiomyosarcomas (MIM: 150800; Tomlinson *et al*, 2002). Fumarate hydratase which is a component of the tricarboxylic acid cycle, acts as a tumour suppressor, its activity being very low or absent in tumours from individuals with leiomyomatosis (Tomlinson *et al*, 2002).

To evaluate the role of *FH* in sporadic leiomyomas and leiomyosarcomas, we analysed 129 uterine leiomyomas and 26 leiomyosarcomas for somatic mutations and allelic imbalance at 1q42.3-q43.

## MATERIALS AND METHODS

### Tumour samples

A series of 129 uterine leiomyomas (89 fresh and 40 paraffin embedded samples) from 21 unselected patients (aged 37 to 50 years) were studied. Number of tumours per patient ranged from one to 14 (average number six). Twenty-six fresh frozen leiomyosarcomas were studied. All were extra-uterine tumours. Histopathology of all tumours was re-examined, and confirmed as soft tissue leiomyosarcomas.

### DNA extraction

Tissues were micro-dissected and digested with 10 mM Tris-HCl (pH 7.5), 1 mM EDTA, 15% (w v^−1^) SDS, and 500 μg ml^−1^ proteinase K, for 16 h at 56°C. DNA was precipitated with sodium acetate and ethanol following phenol chloroform extraction.

### Allele imbalance

The microsatellite markers D1S3462, D1S235, D1S2850, D1S2785, D1S304, D1S180, D1S204, D1S547, D1S1634, D1S1609 were used to evaluate allelic imbalance within the 13.2 Mb region between 1q42.2 and 1q44 (http://genome.ucsc.edu/goldenpath/aug2001Tracks.html). D1S204 and D1S547 flank *FH*. Forward primers were fluorescently-labelled and PCR-amplified products electrophoresed through 6% denaturing polyacrylamide gels and products detected using ABI 377 DNA sequencers. Results were analysed using Genescan and Genotyper software. Analyses suggestive of LOH were repeated at least once to confirm the results. Allele loss was scored if the area under an allelic peak was reduced to <40% of its original value (compared with the other allele).

### Detection of FH mutations

PCR amplification of the 10 exons and intron–exon junctions, including splice sites of FH, was carried out using the primer pairs detailed in Table 1

**Table 1:**
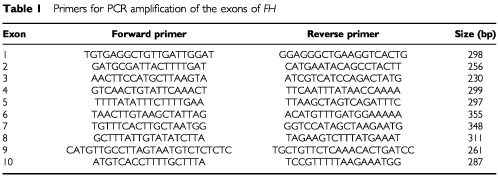
Primers for PCR amplification of the exons of *FH*

. Radio-labelled PCR products were analysed for mutations by conformational sensitive gel electrophoresis (CSGE) as described previously (Ganguly *et al*, 1993). To allow for the detection of sequence variants that may be rendered homozygous in tumours as a result of LOH, heteroduplex formation was carried out after addition of unlabelled PCR product of the identical region from a normal control DNA. Any fragments showing migration shifts were sequenced directly from the PCR products using the ABI Ready Reaction Dye Terminator Cycle Sequencing Kit and an ABI3100 Prism sequencer and analysed using the Sequence Navigator software. Nucleotide changes identified were coded according to the genomic nucleotide sequence at Genbank (http://www.ncbi.nlm.nih.gov/Genbank/) accession number NT_004771.

## RESULTS

The FH gene is composed of 10 exons. The first exon encodes a signal peptide facilitating mitochondrial import of the expressed protein. We screened the full coding sequence and splice junctions of FH in all of the leiomyomas and leiomyosarcomas. Three nucleotide sequence changes were identified. The first was IVS3-22A>T, identified in one leiomyosarcoma. The second was a synonymous substitution – 309C>T encoding alanine identified in one leiomyoma, which was also detected in the germline. The third change was a synonymous substitution – 927G>A encoding proline, identified in two leiomyosarcomas. No missense changes or pathogenic mutations – truncating or splice site – were detected in any of the tumours.

Thirteen of the leiomyosarcomas were assessed for allele imbalance, seven showed LOH (54%). In all the cases allele imbalance was not confined to the vicinity of FH but involved the whole region analysed. There was no evidence from the LOH data for the existence of small-scale deletions in the eight other cases. Chromosome 1q42-q43 imbalance was rare in uterine leiomyomas, only seven of the 129 (5%) tumours displayed LOH.

## DISCUSSION

Prompted by the predisposition to leiomyomata and leiomyosarcoma seen in carriers of germline mutations in FH we have screened the full coding sequence and splice junctions of FH for somatic mutation in a series of sporadic leiomyomas and leiomyosarcomas. Although we cannot exclude the possibility that some mutations may have been missed or cannot be detected by a PCR-based approach, the results suggest that mutations in this gene do not play a major role in the genesis of sporadic forms of these smooth muscle tumours. Although in the leiomyosarcomas LOH was common, this reflected extensive loss of chromosome 1q rather than being limited to the vicinity of *FH*.

The tumour suppressor gene/recessive oncogene hypothesis articulated by Knudson (1993) predicts that genes that confer an increased risk of neoplasms as a result of germline mutations are likely to be somatically mutated in sporadic cancers of the same type. This has proved to be the paradigm of the *RB1* gene and for several other genes, such as *APC*, *NF2* and *TP53*. *FH* does not appear to conform to this model and is similar to a number of other cancer susceptibility genes in this respect. For example, truncating mutations in *BRCA1* and *BRCA2* confer a high risk of breast cancer, but rarely have somatic mutations in sporadic tumours been reported in either gene (Futreal *et al*, 1994; Lancaster *et al*, 1996; Teng *et al*, 1996). Similarly germline mutations in *PTEN* confer an elevated risk of breast cancer in individuals with Cowden's disease, but somatic mutations in *PTEN* are rarely observed in breast cancers outside this context (Rhei *et al*, 1997; Sakurada *et al*, 1997).

There are several possible reasons for the low frequency of FH mutations in sporadic leiomyomas and leiomyosarcomas detected in our study. It may be that sporadic lesions develop through a route distinct from familial tumours. Alternatively, FH expression is altered in ways other than somatic mutation, such as regulation of mRNA levels. To date there are no studies to our knowledge that suggest this is a possibility either in sporadic leiomyomas or leiomyosarcomas. We are, however, in the process of evaluating this possibility.

The mechanism by which FH can act as a tumour suppressor is unclear. It is possible that hypermutability results from oxidative damage. Alternatively, accumulation of fumarate leads to feedback effects on oxidative metabolism and thus on the cell cycle. Smooth muscle cells harbouring ‘two hits’ in *FH* would exhibit inhibition of normal progression of the cell cycle, causing hyperproliferation or a failure of apoptosis. Feedback from fumarate would have tissue-specific effects, hence the growth of smooth muscle tumours (Tomlinson *et al*, 2002). Although mutations in *FH* do not appear to be involved in the development of sporadic smooth muscle tumours it is conceivable that other mitochondrial defects may be involved.
